# Prevalence of frailty and cognitive impairment in older transplant candidates - a preview to the Kidney Transplantation in Older People (KTOP): impact of frailty on outcomes study

**DOI:** 10.1186/s12882-022-02900-w

**Published:** 2022-08-13

**Authors:** Amarpreet K. Thind, Annabel Rule, Dawn Goodall, Shuli Levy, Sarah Brice, Frank J. M. F. Dor, Nicola Evans, David Ospalla, Nicola Thomas, David Wellsted, Lina Johansson, Michelle Willicombe, Edwina A. Brown

**Affiliations:** 1 Department of Immunology and Inflammation, Centre for Inflammatory Disease, Imperial College London, Hammersmith Campus, Du Cane Road, London, W12 0NN UK; 2 Imperial College Renal and Transplant Centre, Imperial College Healthcare NHS Trust, Hammersmith Hospital, Du Cane Road, London, W12 0HS UK; 3 Central London Community Healthcare NHS Trust, Ground Floor, 15 Marylebone Road, London, NW1 5JD UK; 4 Department of Surgery and Cancer, Imperial College London, South Kensington Campus, London, SW7 2AZ UK; 5 Guy’s and St Thomas’ NHS Foundation Trust, Great Maze Pond, London, SE1 9RT UK; 6 Institute of Health and Social Care, London South Bank University, 103 Borough Road, London, SE1 0AA UK; 7 The Centre for Health Services and Clinical Research, The University of Hertfordshire, College Lane, Hatfield, Hertfordshire, AL10 9AB UK

**Keywords:** Kidney transplantation, Older people, Frailty, Cognition

## Abstract

**Background:**

Kidney transplantation in older people has increased, however older transplant recipients experience mixed outcomes that invariably impacts on their quality of life. The increased vulnerability of older end stage kidney disease patients to frailty and cognitive impairment, may partially explain the differences in outcomes observed.

The Kidney Transplantation in Older People (KTOP): impact of frailty on clinical outcomes study is an active clinical study aiming to explore the experience of older people waiting for and undergoing transplantation. In this manuscript we present the study protocol, the study cohort, and the prevalence of frailty and cognitive impairment identified at recruitment.

**Methods:**

The KTOP study is a single centre, prospective, mixed methods, observational study. Recruitment began in October 2019. All patients aged 60 or above either active on the deceased donor waitlist or undergoing live donor transplantation were eligible for recruitment. Recruited participants completed a series of questionnaires assessing frailty, cognition, and quality of life, which are repeated at defined time points whilst on the waitlist and post-transplant. Clinical data was concurrently collected. Any participants identified as frail or vulnerable were also eligible for enrolment into the qualitative sub-study.

**Results:**

Two hundred eight participants have been recruited (age 60–78). Baseline Montreal Cognitive Assessments were available for 173 participants, with 63 (36.4%) participants identified as having scores below normal (score < 26). Edmonton Frail Scale assessments were available for 184 participants, with 29 participants (15.8%) identified as frail (score ≥ 8), and a further 37 participants (20.1%) identified as being vulnerable (score 6–7).

**Conclusion:**

In the KTOP study cohort we have identified a prevalence of 36.4% of participants with MoCA scores suggestive of cognitive impairment, and a prevalence of frailty of 15.8% at recruitment. A further 20.1% were vulnerable. As formal testing for cognition and frailty is not routinely incorporated into the work up of older people across many units, the presence and significance of these conditions is likely not known. Ultimately the KTOP study will report on how these parameters evolve over time and following a transplant, and describe their impact on quality of life and clinical outcomes.

## Background

The end stage kidney disease (ESKD) population is ageing with older people now representing the age group with the highest incidence of ESKD [1]. Traditionally, dialysis modalities have dominated kidney replacement therapy in this age group. However, with growing expectations and increased acceptance of kidney transplantation (KT), rates in older people have also steadily increased [2, 3]. Outcomes of KT in older people are mixed and differ to those observed in younger recipients [4]. Although increases in life expectancy with KT have been extensively reported, the quantity of life years gained decreases with increasing age. Alongside this older KT recipients experience increased peri-operative morbidity, post-transplant infections and prolonged hospitalisation [4–8]. Consequently, the impact of KT on the quality of life of older people is highly variable [9, 10].

At all ages, chronic kidney disease and ESKD populations are more susceptible to developing frailty and cognitive impairment when compared to the general population [11–13]. Frailty is a multidimensional syndrome which results from progressive and sustained degeneration in several physiological systems [11, 14]. It characterises the differences in physiological and chronological age and produces a spectrum of deficits with confer an increased susceptibility to physical stressors and an increased risk of adverse outcomes [11, 12, 14]. Frailty has been reported to affect anywhere between 14 and 73% of adult dialysis dependent patients, and cognitive impairment present in up to 80% [12, 15]. The presence of frailty and cognitive impairment is well recognised as impacting on all aspects of KT, from likelihood of being waitlisted and receiving a KT, through to hospitalisation, patient, and graft survival following a KT [12, 16, 17]. With ageing being well-recognised as a contributing factor to developing both frailty and cognitive impairment, older people with ESKD are particularly vulnerable to the presence of these conditions and the impact they may have on KT progress and success [11–13]. Only more recently have both Kidney Disease Improving Global Outcomes (KDIGO) and the European Renal Association – European Dialysis and Transplant Association highlighted the need for a more tailored assessment of older kidney transplant candidates, with a specific focus on assessing frailty and cognition in order to optimise candidate selection and improve outcomes in the wider context of candidates’ needs [6, 18]. Currently, many transplant units do not routinely incorporate assessments of frailty or cognition as part of the work-up for older candidates, and so the true burden of these syndromes in older people put forward for KT may not be known. Under recognition of these conditions leaves both patients and transplant units underprepared and vulnerable to the adverse outcomes known to occur in frail and/or cognitively impaired KT recipients [12, 15, 17].

In a cohort where KT is increasing, there is a greater vulnerability to frailty and cognitive impairment, which may confer more specific needs. Previous qualitative enquiry has demonstrated that older people experience discrepancy between their expectations of transplant and the reality of adapting to life with a KT [19]. They also report worsening forgetfulness, ability to self-manage, disillusionment with symptoms and increased need for support post-KT [19–21]. However, a longitudinal understanding of being on the waitlist and the transplant experiences of older people is essential to understanding how clinical and experiential outcomes can be optimised in this age group. The Kidney Transplantation in Older People (KTOP): impact of frailty on outcomes study, plans to address this question. The aim of this paper is to present an overview of the KTOP study protocol and describe the study cohort at recruitment.

## Methods

The KTOP study is a single centre, prospective, mixed methods study being conducted at the Imperial College Renal and Transplant Centre (ICRTC) in West London, UK. The investigator team consists of multi-disciplinary colleagues, including nephrologists, transplant surgeons, geriatricians, nurse specialists, pharmacists, dietitians, occupational therapists, and a KT recipient, which has enabled a holistic study design. The KTOP study consists of two concurrently running components, an observational study and a qualitative study.

### KTOP observational study

The observational study began in October 2019 and will continue until June 2023. Favourable ethical approval was received from Yorkshire and the Humber Leeds West Research Ethics Committee and Health Research Authority (REC reference 19/YH/0287). Local institutional review and approval were also obtained.

All patients under the care of ICRTC aged 60 or over and being worked up for KT (living or deceased donor transplantation) or ‘active’ on the national KT waiting list, were eligible for inclusion in the study. Patients with significant language barriers that could not be easily overcome using family members or carers as interpreters, were excluded as they would not be able to engage with study activities to the required level of detail. All potential participants were approached about recruitment to the study when attending for routine healthcare encounters (e.g. haemodialysis sessions or outpatient clinic appointments). On recruitment to the study all study activities were completed at subsequent scheduled healthcare encounters, which limited disturbances to participant’s personal time. Informed written consent was obtained from all participants recruited into the study. Following recruitment all participants completed a set of baseline questionnaires (Table 1), which were completed prior to KT.

**Table 1:** Summary of KTOP Observational Study Questionnaires

Study Questionnaires	
Montreal Cognitive Assessment	Short Form −12 version 2
Edmonton Frail Scale	Depression Patient Health Questionnaire-9
Subjective Global Assessment of Nutrition	Illness Intrusiveness Scale
Nottingham Activities of Daily Living Scale	Renal Treatment Satisfaction Questionnaire
Social Support Questionnaire	Palliative Care Outcome Scale – Renal
Beliefs About Medications Questionnaire	^a^Basel Assessment of Adherence to Immunosuppression

Each questionnaire is completed with participants at every study visit. ^a^The Basel Assessment of Adherence to Immunosuppression questionnaire is completed by transplant recipients only during their post-transplant study visits

**Fig. 1:**
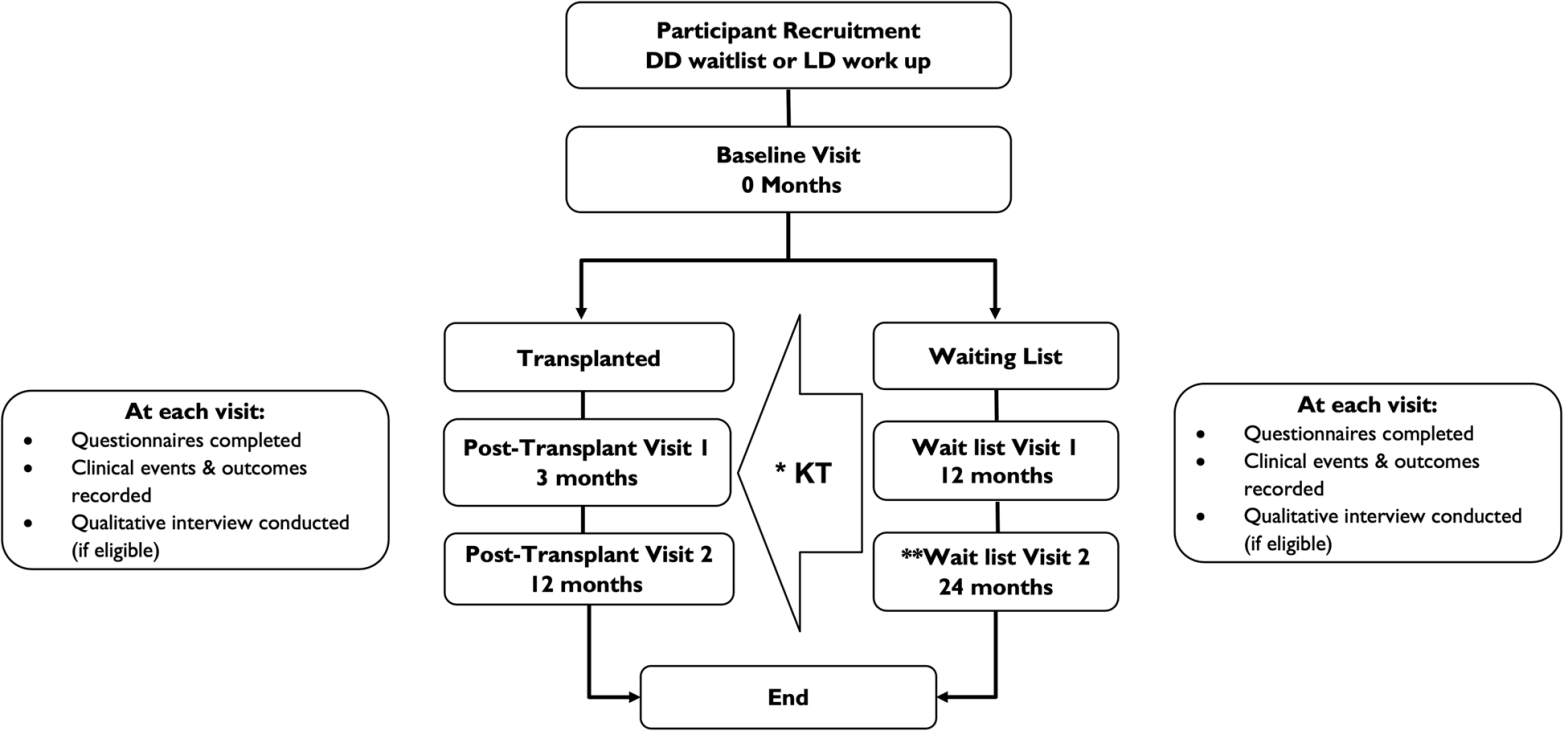
Overview of study visits and activities. This diagram summarises the journey of a participant through the KTOP study, including the timing of study visits and the activities completed at each visit. *At any time during the study a participant may move from the waitlist to receiving a transplant. The timing of follow up visits are adjusted accordingly. **Waitlist visit 2 is applicable to participants in the observational study only. DD – deceased donor, LD – liver donor, KT – kidney transplantation

These questionnaires were then repeated either annually for 2 years in those participants who remain on the waitlist, or at 3- and 12-months post-transplantation in those participants who were transplanted during the study period (Fig. 1). The questionnaires assess frailty, cognition, nutritional status, functional status, social support, quality of life and medication management. Each questionnaire was chosen in collaboration with and based on recommendations from the multi-disciplinary investigator group, and each has been validated for use in people with chronic diseases [22–31]. Alongside completion of the questionnaires, demographic, medical history, clinical event, and outcome (survival, graft function) data were concurrently collected at the defined time points.

The immune system in older people may be altered by both ageing and the presence of frailty [12, 32]. CD8 and CD4 lymphocyte ratios have been correlated with immune risk in KT recipients, and changes in this ratio are recognised to occur in older people over time [33, 34]. For all recruited participants a whole blood ethylenediaminetetraacetic (EDTA) sample was also collected to measure lymphocyte subsets. These results will be used to determine the association of lymphocyte subsets with frailty assessments and clinical outcomes.

The unpredictable nature of deceased donor KT meant 24 participants were transplanted prior to recruitment into the study. To maximise participation these individuals were still approached and recruited into the study but only completed the post-KT assessments, and did not have serum collected as the use of induction agents at the time of KT would have affected lymphocyte subset levels.

### KTOP qualitative study

The descriptive qualitative study began in April 2021 and will continue until April 2023. Favourable ethical approval was received from London Stanmore Research Ethics Committee and Health Research Authority (REC reference 20/LO/1208), and local approval from the institutions research office. All participants on the KT waitlist who were identified as frail or vulnerable from the Edmonton Frail Scale (EFS) assessment (score of ≥6), were also eligible for recruitment to the adjacent KTOP qualitative study. This is being conducted by a subgroup within the KTOP investigator team and aims to explore the lived experiences of older people vulnerable to frailty, whilst on the waitlist and following a KT, using a variety of data collection methods. The qualitative study is using semi-structured interviews at defined time points on the waitlist (0 and 12 months) and following a KT (3 and 9–12 months) to explore the experiences in more detail. These interviews will be analysed using Thematic Analysis [35].

In addition to the interviews and to ensure a broader approach to participants’ communication needs, the option to complete a patient diary was provided, to triangulate with the data obtained from the study interviews.

This paper will focus on presenting the results from the baseline assessments for all participants recruited to the KTOP observational study, prior to KT. As the KTOP study remains active and will continue until 2023, the wider results of the study will be available in subsequent publications following completion of the study. Descriptive statistics were calculated for the results presented here, and Chi-squre, Fisher’s Exact or t-tests were used for appropriate group comparisons. A two-sided level of significant was set at *p* < 0.05. All analyses were completed using Stata/BE version 17.0 (StataCorp LLC, Texas), with advisory support from the University of Hertfordshire.

## Results

Two hundred and eight patients have been recruited into the KTOP study since October 2019, 184 (88.4%) of whom were recruited prior to transplantation and have completed baseline assessments. The median age at recruitment was 65 years old (range 60–78), with 66% of the cohort being male. Table 2 summarises the demographics of the study participants at the point of recruitment.

**Table 2:** Demographic characteristics of the observational study cohort

Characteristic		Count (***n*** = 208)
**Age (median, range) (years)**		65 (60–78)
**Male**		137 (65.9)
**Ethnicity**	**South Asian**	96 (46.2)
	**Caucasian**	55 (26.4)
	**Afro-Caribbean**	33 (15.9)
	**Middle Eastern**	14 (6.7)
	**East Asian**	10 (4.8)
**Cause of ESKD**	**Diabetes**	94 (45.2)
	**Unknown**	28 (13.5)
	**Glomerulonephritis**	23 (11)
	**PKD**	16 (7.7)
	**Urological**	14 (6.7)
	**Other**	12 (5.8)
	**Renovascular disease**	9 (4.3)
	**Hypertension**	7 (3.4)
	**FSGS**	5 (2.4)
**Modality**	**ICHD**	170 (81.7)
	**Home haemodialysis**	1 (0.5)
	**Peritoneal Dialysis**	25 (12)
	**AKCC**	12 (5.8)
**Mean KRT Vintage (days) (LQ-UQ)**		1011 (307–1340)
**Previously Transplanted**		39 (18.8)
**Charlson Co-morbidity index score (mean, LQ - UQ)**		6.0 (5–7)
**Nottingham Activity of Daily Living score (median, range)**		17 (2–22)
**Depressive Symptoms Present**		73 (39.7)
**Educational Age (median, range)**		18 (0–40)

Results presented as n (%) unless otherwise stated. *ESKD* End stage kidney disease, *PKD* Polycystic kidney disease, *FSGS* Focal segmental glomerulosclerosis, *ICHD* In centre haemodialysis, *AKCC* Advanced kidney care clinic, *KRT* Kidney replacement therapy, *LQ* Lower quartile, *UQ* Upper quartile

### Prevalence of cognitive impairment

A baseline Montreal Cognitive Assessment (MoCA) score was available in 173 patients. A MoCA assessment was not available on all participants due to the presence of learning difficulties or in some cases limitations to their English language skills. In these cases, although participants may have sufficient levels of English to engage with many of the other questionnaires, because of the familiarity with English required to complete the MoCA accurately, their English language skills were not always sufficient for this and so a MoCA was not performed in these participants. The results presented here represent MoCA scores for participants at the point of recruitment to the study and are therefore prior to KT. The mean MoCA score was 25.96 (SD 3.12, 95% CI 25.49–26.43). Sixty-three participants (36.4%) were found to have scores suggestive of cognitive impairment (defined as a MoCA score < 26), whilst 110 participants (63.6%) had normal MoCA scores. A MoCA score cut-off of < 26 was used as the threshold suggestive of cognitive impairment in this study, as this is in keeping with existing practices within our unit and is the cut off suggested by the MoCA tool itself. However, it must be recognised that debate does exist around the optimal MoCA score that adequately detects mild cognitive impairment with improved specificity and sensitivity [36]. Table 3 summarises the demographic characteristics between the patients with no suggestion of cognitive impairment and those with suggested cognitive impairment. The demographics found to be significantly different between those with suggested cognitive impairment and without, were ethnicity (*p* < 0.001), modality of treatment (*p* = 0.023), and lower activities of daily living scores (*p* = 0.0016), lower educational age (*p* = 0.0212) and a higher presence of depressive symptoms (*p* = 0.034) as described in Table 3.

**Table 3:** Demographic characteristics of study cohort by degree of suggested cognitive impairment

Characteristic		No Cognitive impairment ***N*** = 110 (%)	Cognitive Impairment ***N*** = 63 (%)	***P*** value
**Age (mean, range) (years)**		65.2 (60–78)	66.1 (60–77)	0.194
**Gender**	**Male**	73 (66.4)	41 (65.1)	0.864
	**Female**	37 (33.6)	22 (34.9)	
**Ethnicity**	**South Asian**	41 (37.3)	40 (63.5)	< 0.0001
	**Caucasian**	39 (35.5)	6 (9.5)	
	**Afro-Caribbean**	21 (19.1)	8 (12.7)	
	**Middle Eastern**	4 (3.6)	6 (9.5)	
	**East Asian**	5 (4.6)	3 (4.8)	
**Modality**	**ICHD**	86 (78.2)	57 (90.48)	0.023
	**Home-HD**	0	1 (1.6)	
	**PD**	17 (15.5)	5 (8)	
	**AKCC**	7 (6.4)	0	
**KRT Vintage (mean, LQ-UQ)**		993 (200–1301)	1000 (481–1344)	0.9703
**Previously transplanted**		26 (23.6)	9 (14.3)	0.141
**Charlson Co-morbidity Index score (mean, LQ-UQ)**		5.8 (5–7)	6.2 (5–8)	0.07
**Diabetes**		54 (49.1)	40 (63.5)	0.067
**Peripheral Vascular Disease**		5 (4.6)	1 (1.6)	0.418
**Hypertension**		90 (81.8)	58 (92.1)	0.065
**Ischaemic Heart Disease**		48 (43.6)	33 (52.4)	0.267
**Cerebrovascular Accident**		13 (11.8)	11 (17.5)	0.362
**Nottingham Activities of Daily Living Score (mean, range)**		17 (2–22)	14.4 (4–21)	0.0016
**Depressive symptoms present**		41 (37.6)	31 (50.8)	0.034
**Educational age (mean, range) (years)**		19.2 (12–40)	17.4 (0–36)	0.0212

Results presented as n (%) unless otherwise stated. *KRT* Kidney replacement therapy, *ICHD* In centre haemodialysis, *HD* Haemodialysis, *PD* Peritoneal dialsysis, *AKCC* Advanced kidney care clinic, *LQ* Lower quartile, *UQ* Upper quartile. *P* values calculated using t-tests for continuous variables, and Chi-sqare or Fisher’s exact testing for categorical variables (determined by the number of observations per group)

**Fig. 2:**
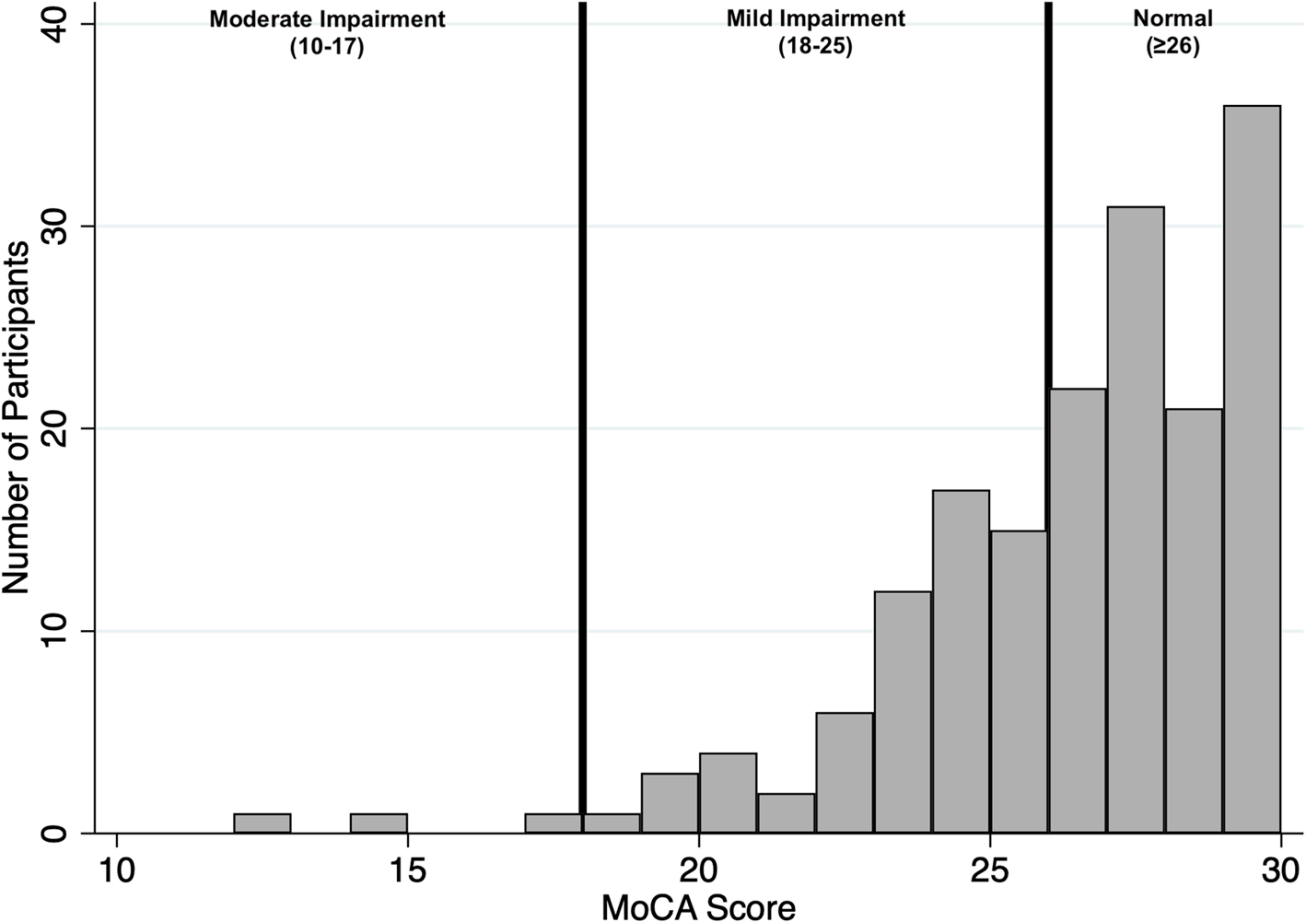
Histogram of MoCA scores and the associated degree of cognitive﻿ impai﻿rment. This histogram illustrates the frequency and distribution of Montreal Cognitive Assessment (MoCA) scores across the cohort. The vertical black lines represent the MoCA score boundaries which suggest the degree of cognitive impairment the MoCA scores correspond to. Normal cognition is a MoCA score of ≥26, mild impairment is a score of 18–25, and moderate impairment is a score of 10–17

In those patients suggested as having cognitive impairment most patients (60) had mild cognitive impairment (MoCA score 18–25), with only 3 patients with scores suggestive of having moderate cognitive impairment (MoCA score 10–17) at recruitment. The distribution of MoCA scores within the KTOP study cohort and how these scores translate into possible degrees of cognitive impairment, are summarised in Fig. 2.

### Prevalence of frailty

The Edmonton Frail Scale (EFS) was chosen as a it is a well validated and reliable frailty tool, that is easy to perform without requiring specific training. This was appropriate to this study, as multiple questionnaires were being conducted by the same person (study researchers). Furthermore, the EFS assesses several components of frailty, which provides an opportunity to identify components that could be targeted by specific intervention. EFS scores were available for 184 patients. The mean EFS score was 4.9 (SD 2.51, 95% CI 4.5–5.2) across the study cohort. One hundred and eighteen participants (64.13%) were identified as being ‘not frail’ based on their EFS scores, whilst 29 participants (15.8%) were identified as ‘frail’ (EFS score of ≥8), with a further 37 participants (20.1%) identified as being ‘vulnerable’ (EFS score 6–7). The demographic characteristics of the not frail and vulnerable/frail groups are presented in Table 4. Significant differences between the not frail and vulnerable/frail groups were observed in relation to ethnicity (*p* < 0.0001), modality of treatment (*p* = 0.012), mean Charlson comorbidity index score (*p* = 0.010), the presence of diabetes (*p* = 0.003), peripheral vascular disease (*p* = 0.023), depressive symptoms *(p* < 0.0001), mean activities of daily living scores (*p* < 0.0001) and educational age (*p* = 0.007) (Table 4).

**Table 4:** Demographic characteristics of study cohort by frailty status

Characteristic		Not-Frail ***N*** = 118 (%)	Frail/Vulnerable ***N*** = 66 (%)	***P*** value
**Age (mean, range) (years)**		66 (60–77)	65 (60–78)	0.051
**Gender**	**Male**	78 (66.1)	42 (63.6)	0.736
	**Female**	40 (33.9)	24 (36.4)	
**Ethnicity**	**South Asian**	57 (48.3)	28 (42.4)	< 0.0001
	**Caucasian**	38 (32.2)	9 (13.6)	
	**Afro-Caribbean**	16 (13.6)	15 (22.7)	
	**Middle Eastern**	2 (1.7)	10 (15.2)	
	**East Asian**	5 (4.2)	4 (6.1)	
**Modality**	**ICHD**	91 (77.1)	62 (93.9)	0.012
	**Home-HD**	1 (0.9)	0	
	**PD**	19 (16.1)	4 (6.1)	
	**AKCC**	7 (5.9)	0	
**KRT Vintage (mean, LQ-UQ) (days)**		918 (197–1214)	1141 (476–1468)	0.2146
**Previously transplanted**		21 (17.8)	15 (22.7)	0.419
**Charlson Co-morbidity Index score (mean, LQ-UQ)**		5.8 (4–7)	6.4 (5–7)	0.010
**Diabetes**		55 (46.6)	46 (69.7)	0.003
**Peripheral Vascular Disease**		1 (0.9)	5 (7.6)	0.023
**Hypertension**		100 (84.8)	59 (89.4)	0.377
**Ischaemic Heart Disease**		53 (44.9)	35 (53)	0.291
**Cerebrovascular Accident**		15 (12.7)	11 (16.7)	0.460
**Nottingham Activities of Daily Living Score (mean, range)**		17.9 (5–22)	12.6 (2–21)	< 0.0001
**Depressive symptoms present**		30 (25.4)	43 (65.2)	< 0.0001
**Educational age (mean, range) (years)**		19.2 (0–40)	17.1 (0–35)	0.007

Results presented as n (%) unless otherwise stated. *KRT* Kidney replacement therapy, *ICHD* In centre haemodialysis, *HD* Haemodialysis, *PD* Peritoneal dialysis, *AKCC* Advanced kidney care clinic, *LQ* Lower quartile, *UQ* Upper quartile. *P* values calculated using one way t-tests for continuous variables, and Chi-sqare or Fisher’s exact testing for categorical variables (determined by the number of observations per group)

**Fig. 3:**
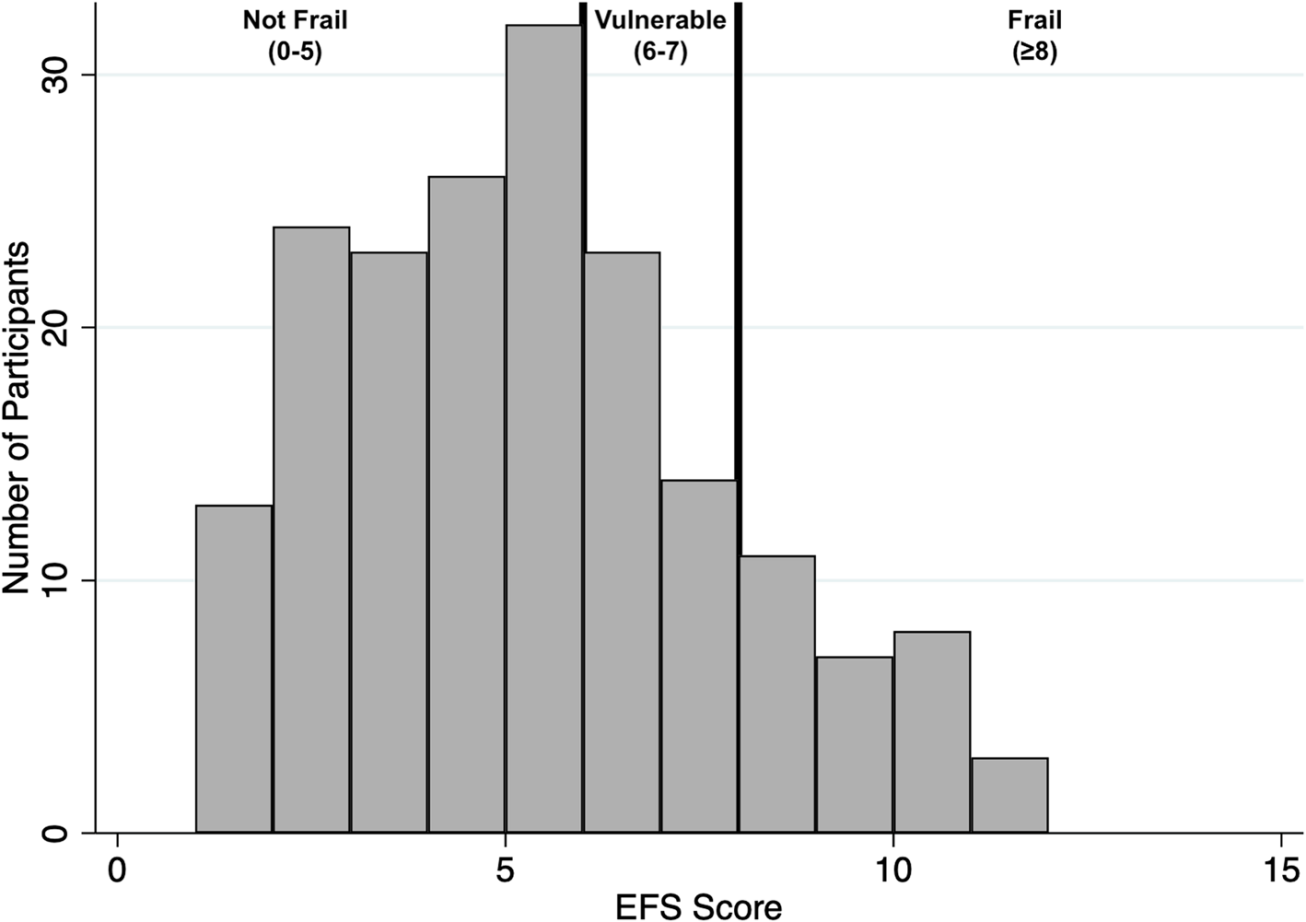
Histogram of EFS scores and the associated frailty status. The histogram illustrates the frequency and distribution of Edmonton Frail Scale (EFS) scores across the cohort. The  vertical black lines represent the EFS score boundaries which the frailty statuses correspond to. Not frail is an EFS score of 0–5, vulnerable is a score of 6–7, and frail is a score of ≥8

Within the group identified as frail (29), 18 participants were defined as having ‘mild frailty’ (EFS score 8–9), 10 participants had ‘moderate frailty’ (EFS score 10–11) and 1 participant was identified as having ‘severe frailty’ (EFS score 12–17). Figure 3 illustrates the distribution of EFS scores across the cohort and how these scores translate into a frailty status.

**Fig. 4:**
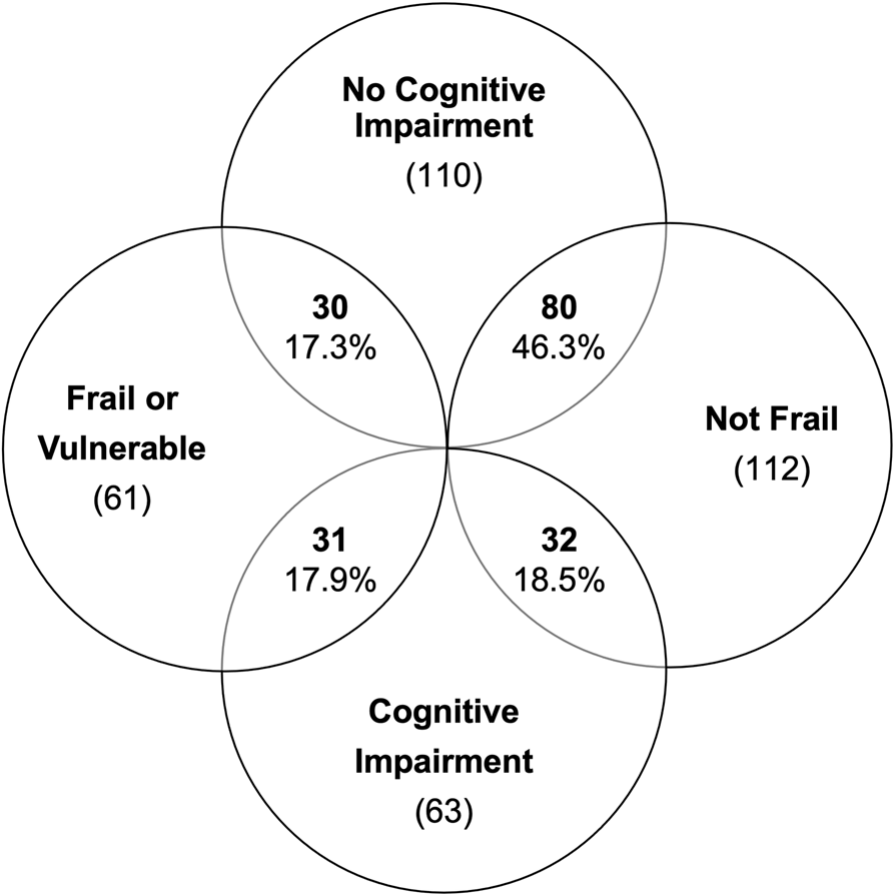
An overview of patients’ identified frailty status and the overlap with suggested cognitive impairment. Total number of patients in each group presented, as well as the proportion (%) of patients overlapping each of the groups

One hundred and seventy-three patients had both MoCA and EFS scores available. In these patients, 80 (46.3%) had neither scores suggestive of the presence of cognitive impairment nor the presence of frailty or vulnerability to frailty. Thirty-one participants (17.9%) were identified as frail or vulnerable and had scores suggestive of cognitive impairment, whilst 30 (17.3%) participants were frail/vulnerable and had normal MoCA scores, and 32 (18.5%) had normal frailty status but MoCA scores suggestive of cognitive impairment. Figure 4 summarises the proportion of patients in each of these groups.

## Discussion

This paper has presented the protocol for the KTOP study and provided details of patient demographics at baseline. The MoCA assessments have suggested a prevalence of cognitive impairment of 36.4% in the KTOP study cohort at the time of recruitment. Although in most cases the degree of cognitive impairment identified was mild, this finding highlights the extent to which abnormal cognition exists in older people being considered for and undergoing KT. The prevalence of frailty in this study cohort at recruitment was identified as 15.8%, with a further 20.1% of the cohort being considered vulnerable. Therefore in combination more than a third of the older people listed for KT at the ICRTC were either vulnerable or frail whilst on the waitlist for KT. In those participants identified as being frail the majority were defined as having ‘mild frailty’ (62%). In those patients where both cognition and frailty was assessed (173 patients), 53.7% were identified as having a degree of deficit related to these syndromes, within which 17.5% were found to have both suggested cognitive impairment and a frail/vulnerable frailty status.

The presence and extent of cognitive impairment and frailty in this cohort is not likely to be known by the local nephrology team, as routine assessments for frailty and cognition in older transplant candidates are not performed at the ICRTC. Many other transplant units may also be under recognising the presence of these conditions, as existing literature has disagreed on which tools should be used for assessing frailty and cognition, and the need to do so in older KT candidates specifically has only recently been recommended by expert groups [3, 6, 17, 18]. Widespread implementation is therefore limited as demonstrated by a survey of KT health professionals where 98.9% of respondents felt that frailty tools would be useful in evaluating KT candidates, but only 23.9% reported to perform this assessment as part of their standard care [37]. Our findings emphasise the subtle and often undetected nature of these deficits in this population. Improved recognition in older KT candidates may allow for better preparation and support (e.g. rehabilitation, social support, medication reviews) for frail or cognitively impaired older people who are navigating the often complicated and lengthy transplant process [17].

The prevalence of cognitive impairment in KT waitlist candidates has been reported as ranging from 5 to 58% in previously studied populations [15, 16, 38]. Gupta and colleagues assessed cognitive impairment and listing for KT in adults (aged 40–68) and found that at initial evaluation 55% of the patients referred had a degree of cognitive impairment present, with 49% having mild impairment and 6% having severe impairment [38]. In those patients who went on to be listed for KT, the prevalence of cognitive impairment was then reported as 23% [38]. The prevalence of cognitive impairment observed in this cohort of 36.4% is therefore in keeping with that reported in wider data. As the KTOP study is focussed on older people it is unsurprising that the prevalence we have observed is at the upper end of the range previously reported across all KT candidates [13, 15, 16, 38]. Our data is also in agreement that in the majority of cases the degree of cognitive impairment identified is mild in nature (95% of KTOP study cohort with cognitive impairment were defined as having a mild impairment).

The prevalence of frailty observed in this study cohort was 15.8%, which is again in agreement with existing literature. A recent systematic review conducted by Quint and colleagues identified a pooled prevalence of frailty of 17.1% in KT candidates based on 14 studies included in the review (KT recipient mean age ranged from 44 to 54 years old) [3]. Across the 14 studies the reported prevalence of frailty ranged from 11.2 to 25.1% [3]. Furthermore, Pérez-Sàez and colleagues identified that in 455 KT candidates, 30% of cohort were found to be pre-frail or frail [39]. This is again similar to our observation that 35.9% of the KTOP cohort were found to be either frail or vulnerable. These finding suggests that in addition to frail participants being put forward for KT, a further proportion of candidates are vulnerable (pre-frail) and may represent a wider group that also requires additional attention.

In this cohort, differences in ethnicity, modality of treatment, activities of daily living scores, the presence of depressive symptoms, and educational age were found to be significant to both the identification of cognitive impairment, and frailty status. Additionally, comorbidity burden, diabetes, and peripheral vascular disease were found to be significantly different across the categories of frailty status, but not cognitive impairment. These characteristics are in keeping with risk factors for frailty and cognitive impairment development reported in existing literature [11, 12, 14, 16]. Subsequent analyses and publications from the KTOP study will determine the association of these factors, and others, on the longitudinal changes in frailty and cognitive function which occur over time, and their impact on clinical and experiential outcomes in this cohort.

Limitations of this work include that this is experience from a single centre, the use of only the English version of the MoCA, and the use of a single tool for assessing frailty and cognition. Although by using only the English version of the MoCA it has meant that some participants with limited English could not complete this assessment, this represents only 6% (12 participants) of the study cohort recruited pre-KT. The estimate of cognitive impairment reported here is therefore still likely to be reliable, as there is little to suggest that cognitive impairment should be higher in those people who do not speak fluent English. Similarly, the use of only a single tool to assess frailty (EFS) and cognition (MoCA) may limit our results, as prior studies have demonstrated disparity in detection of these syndromes when multiple tools are used [18, 40]. Multiple assessments for frailty and cognition were not used to avoid placing a high burden of questionnaires on the participants, and instead maximise their engagement across all other study questionnaires.

Where prior studies have reported on the prevalence of frailty and cognitive impairment across all KT candidates, this paper, and the KTOP study more widely, has focused on older people [3]. This is of particular importance as the older age group are at higher risk of developing these conditions and their presence is likely to have a greater impact on waitlist progress and KT success. Both cognitive impairment and frailty are associated with increased risks of post-operative complications (delirium, prolonged length of stay, mortality, increased functional dependence, increased likelihood of institutionalisation on discharge), therefore failing to recognise these conditions leaves both patients and KT units highly exposed [6, 12, 41]. The latest KT evaluation guidelines from KDIGO recommend performing frailty assessments in older candidates in order to improve risk assessment and identify patients that may benefit from optimisation strategies (e.g. rehabilitation) prior to transplantation. Incorporation of this approach would help ensure that frailty or older age are not seen as barriers to KT.

On completion the KTOP study will report on the longitudinal changes in frailty, cognition, and quality of life for older individuals both over time and pre- and post-KT, as well as how the presence of these conditions impact on clinical and experiential outcomes.

## Conclusion

This study has identified that in people aged 60 years or older who are listed for KT at an urban, renal centre in the UK, over a third (36.4%) had evidence of cognitive impairment present, and over a third were either frail or vulnerable (35.9%), whilst on the waitlist for KT. Ultimately, the KTOP study will provide more detailed holistic information on how older people living with ESKD experience life on the waitlist and following a KT. This will enable a tailored assessment of the older KT candidate, improved risk assessment and communication, and enhanced shared decision making with this vulnerable cohort during a highly dynamic time in their lives.

## Data Availability

The datasets generated and analysed during the current study are not publicly available as the study remains active and so data collection continues. The data reported in this manuscript is available from the corresponding author on reasonable request.

## Abbreviations

KTOP: Kidney Transplantation in Older People
ESKD: End stage kidney disease
KT: Kidney transplantation
KDIGO: Kidney Disease Improving Global Outcomes
ICRTC: Imperial College Renal and Transplant Centre
MoCA: Montreal Cognitive Assessment
EFS: Edmonton Frail Scale
EDTA: Ethylenediaminetetraacetic
